# Supplementary figures and images for: The TRP-channel painless mediates substrate stiffness sensing in the legs during Drosophila oviposition
Source: PLoS Genet. 2025 Dec 29;21(12):e1011980. doi: 10.1371/journal.pgen.1011980 (PMC12768414; doi:10.1371/journal.pgen.1011980)

**A**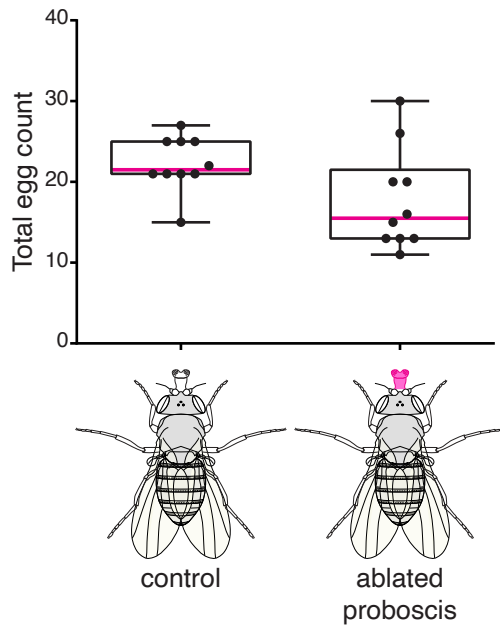**B**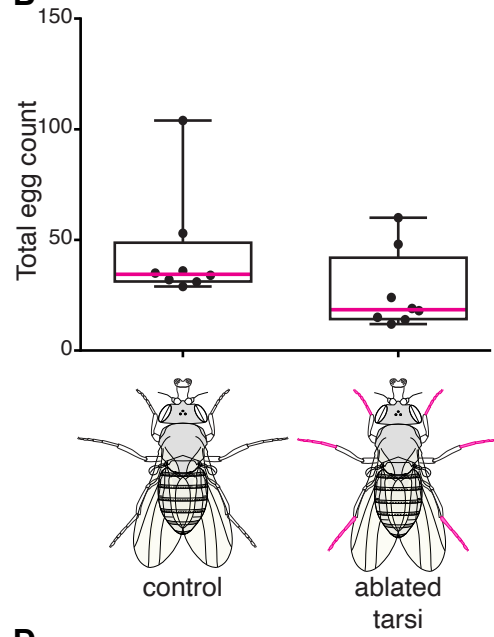**C**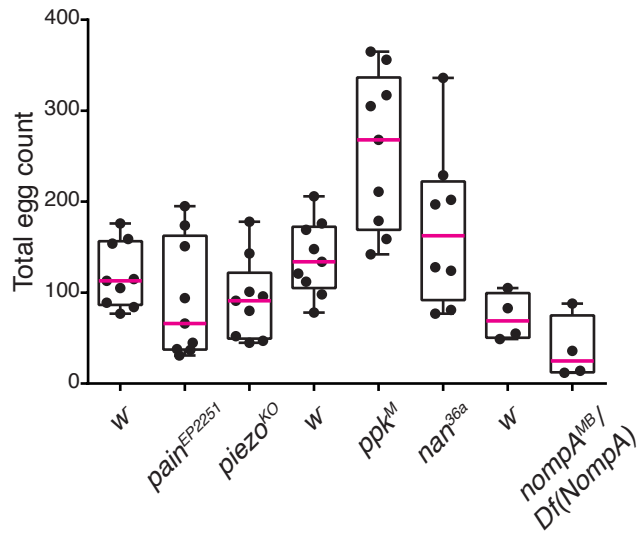**D**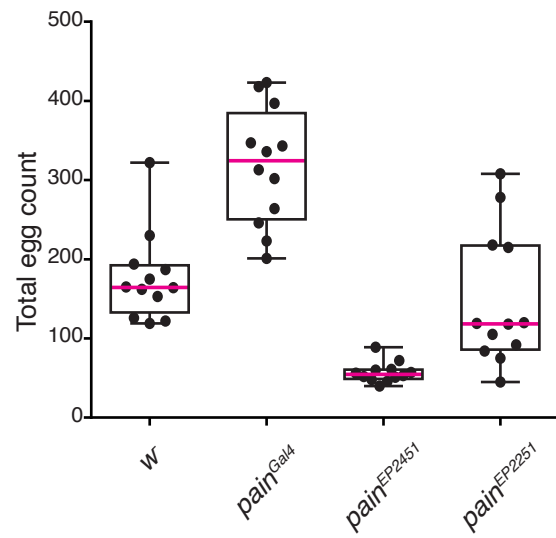**E**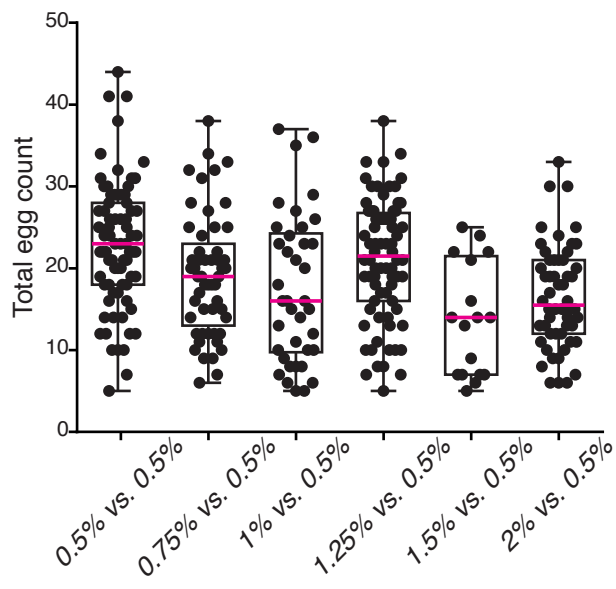**F**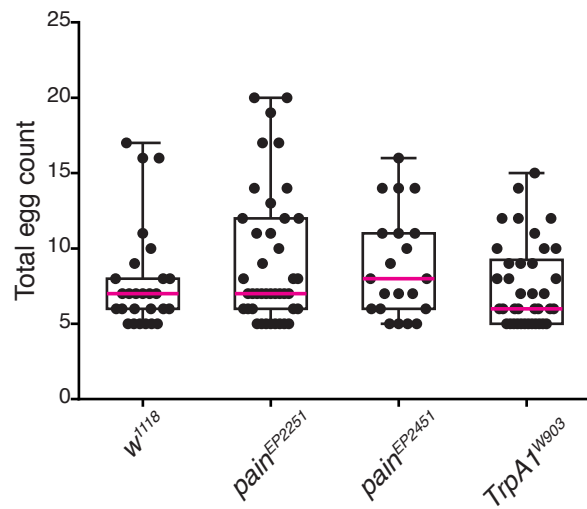

Supplement: S1 Fig — (A-F) Total number of eggs laid per replicate during the group assays involving ablation of proboscis (A) or tarsi (B), as well as genetic screening of potential mechanoreceptors (C) and painless alleles (D). (E-F) Total number of eggs laid per mated female during the single fly assays testing oviposition preferences of wild-type flies involving increasing agarose concentration (E) and mutant alleles of TrpA channels (F). (PDF) [file pgen.1011980.s001.pdf]

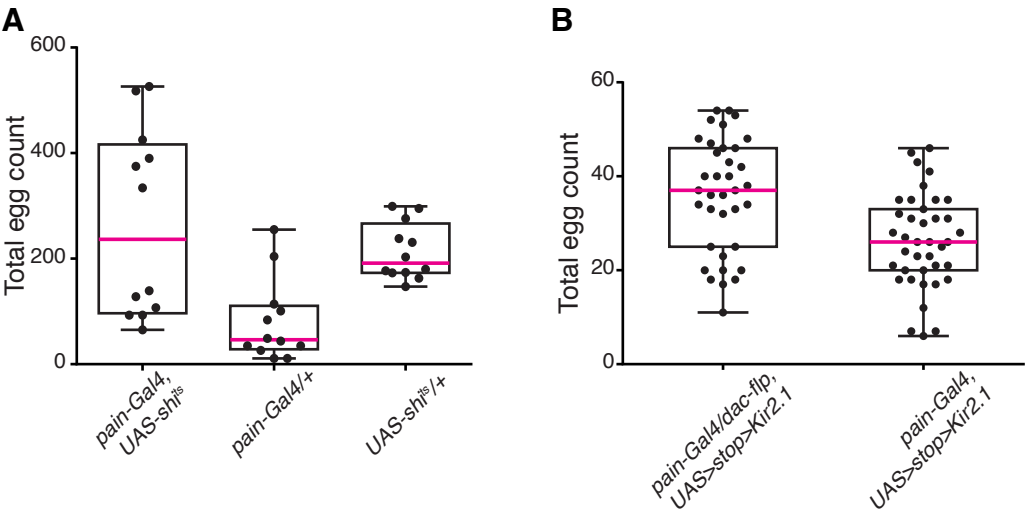

Supplement: S2 Fig — (A-B) Total number of eggs laid per replicate during the group assays, upon silencing of all pain-expressing neurons labelled by pain-Gal4 (A) and per mated female during the single fly assays, involving leg-specific silencing of pain-expressing sensory neurons using dac-flippase (B). (PDF) [file pgen.1011980.s002.pdf]

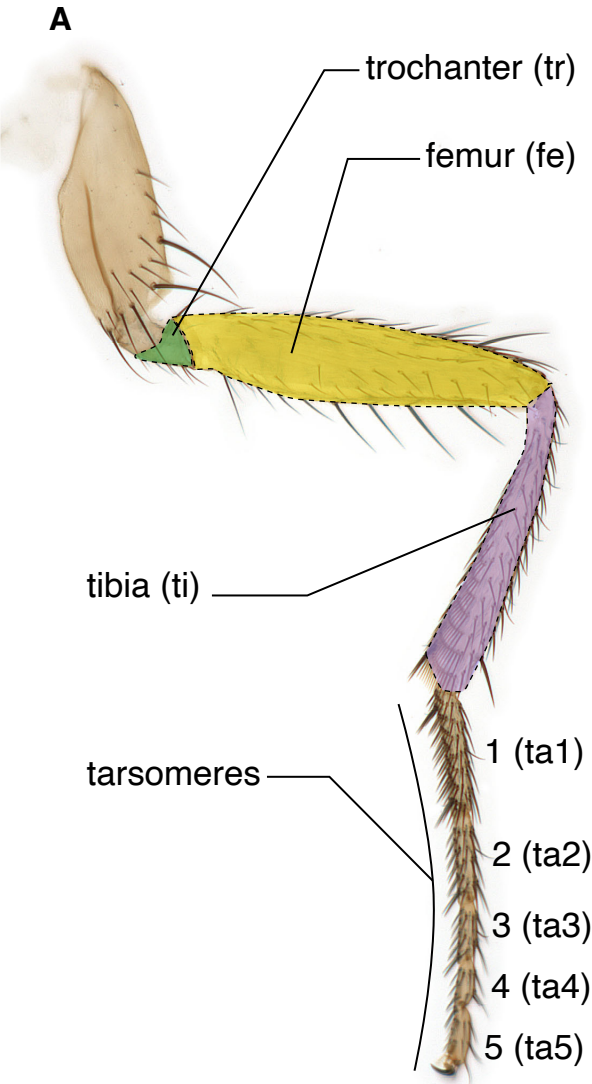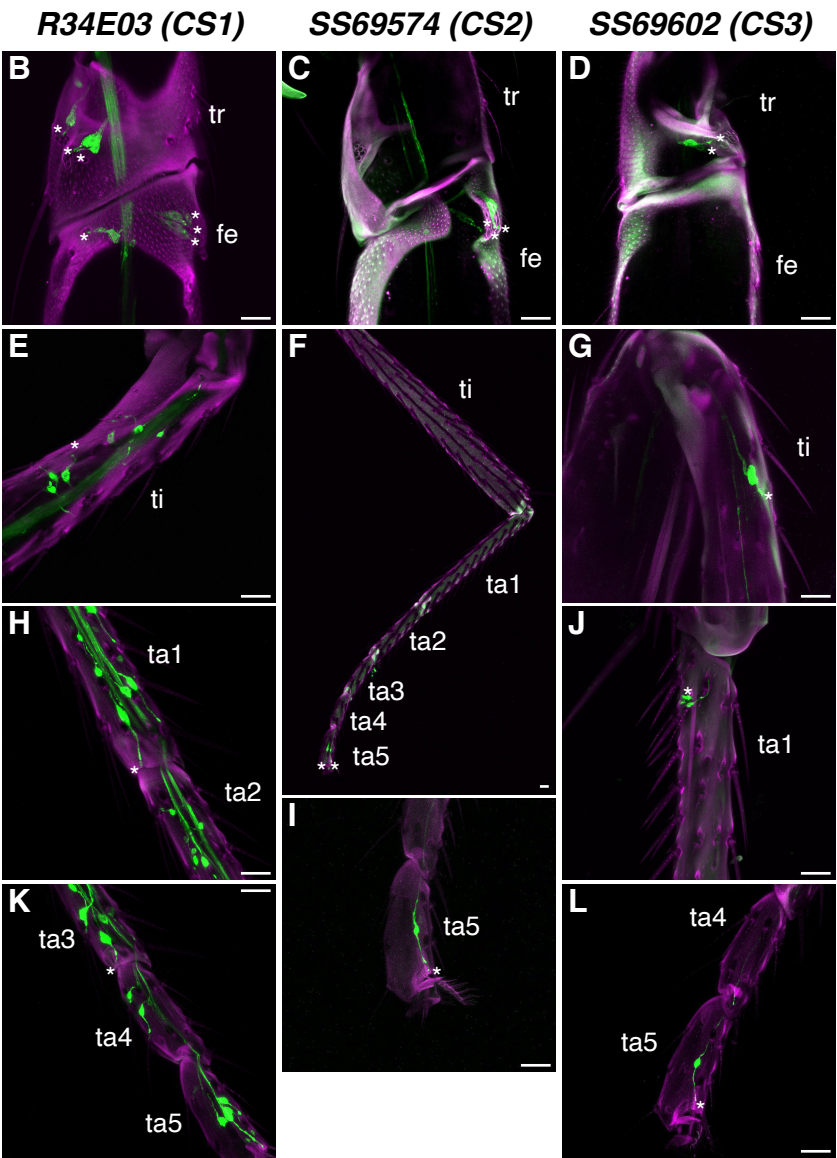

Supplement: S3 Fig — (A) Anatomy of a mesothoracic leg. Confocal images of the expression patterns of CS1-Gal4 (B,E,H,K), CS2-Gal4 (C,F,I) and CS3-Gal4 (D,G,J, L) across the mesothoracic leg. The CS labelled by these driver lines are marked with an asterix (*). Scale bar, 20 µm. (PDF) [file pgen.1011980.s003.pdf]

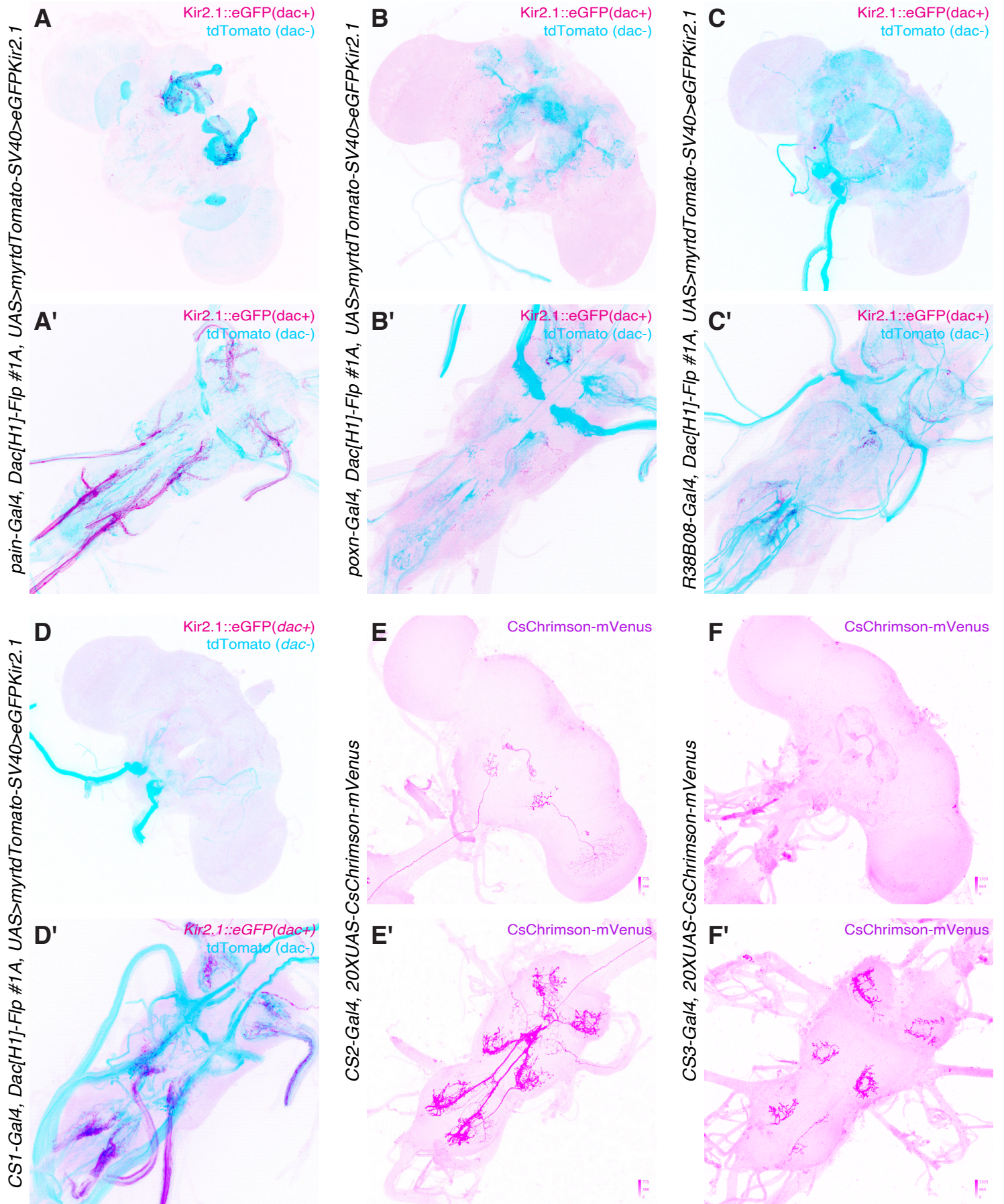

Supplement: S4 Fig — Antibody staining against GFP (Kir2.1-eGFP-expressing Gal4 neurons, magenta) and DsRed (tdTomato-expressing Gal4 neurons, blue) on brains (A-F) and ventral nerve cord (A’-F’) from females with the following genotypes: (A, A’) pain-Gal4, Dac[H1]-Flp, UAS>myrtdTomato-SV40 > eGFPKir2.1. (B, B’) poxn-Gal4, Dac[H1]-Flp, UAS>myrtdTomato-SV40 > eGFPKir2.1. (C, C’) R38B08-Gal4, Dac[H1]-Flp, UAS>myrtdTomato-SV40 > eGFPKir2.1. (D, D’) CS1-Gal4, Dac[H1]-Flp, UAS>myrtdTomato-SV40 > eGFPKir2.1. (E, E’) CS2-Gal4 > 20XUAS-CsChrimson-mVenus. (F, F’) CS3-Gal4 > 20XUAS-CsChrimson-mVenus. (PDF) [file pgen.1011980.s004.pdf]

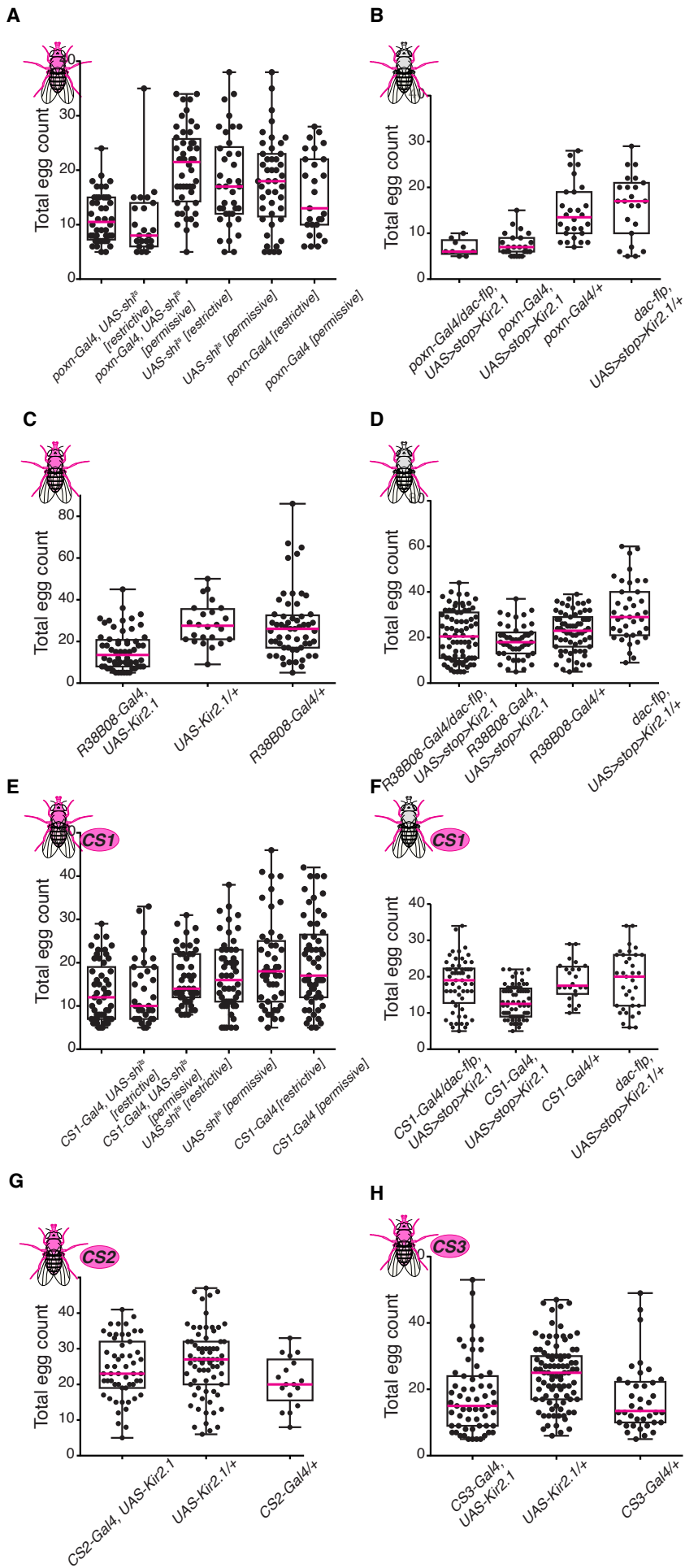

Supplement: S5 Fig — (A-H) Total number of eggs laid per mated female, involving Kir2.1- or Shibirets-mediated silencing of neurons labelled by poxn-Gal4 (A-B), R38B08-Gal4 (C-D) and CS1-Gal4 (E-F), CS2-Gal4 (G), and CS3-Gal4 (H). (PDF) [file pgen.1011980.s005.pdf]

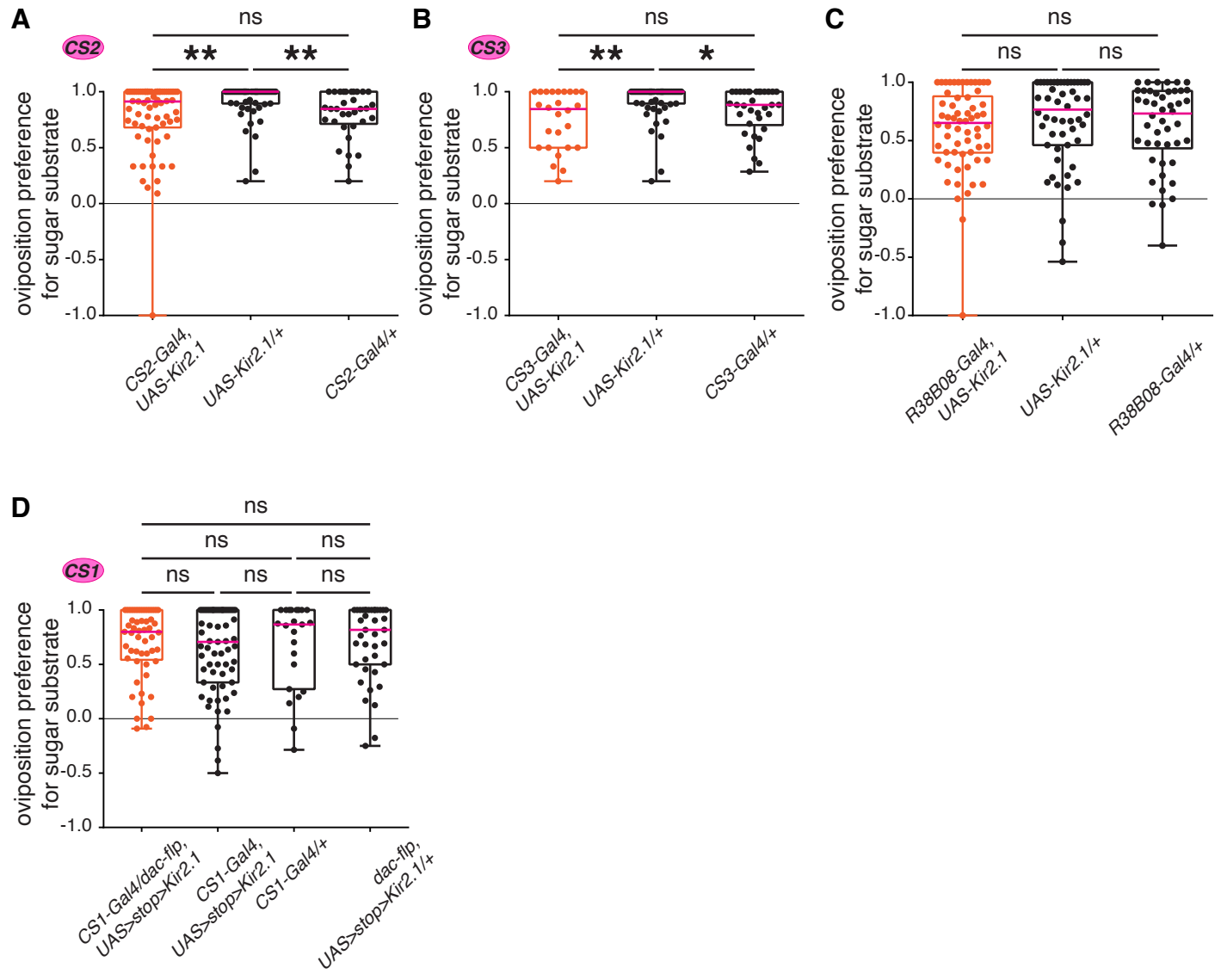

Supplement: S6 Fig — Single-fly two-choice sugar assay (200 mM glucose + 1% agarose vs. 1% agarose) of females with global (A- C) or leg-restricted (D) blocking of neurotransmission in mechanosensory neurons innervating campaniform sensilla (A-B, D) or mechanosensory bristles (C). (A) CS2-Gal4, UAS-Kir2.1, compared to UAS-alone or Gal4-alone controls (n = 67, 53, and 33, respectively). (B) CS3-Gal4, UAS-Kir2.1, compared to UAS-alone or Gal4-alone controls (n = 26, 53, and 34, respectively). (C) R38B08-Gal4, UAS-Kir2.1, compared to UAS-alone or Gal4-alone controls (n = 62, 54, and 46, respectively). (D) conditional inactivation of CS1-Gal4 neurons in the legs, (CS1-Gal4, dac-flippase, UAS>stop>Kir2.1), compared to controls lacking one of the genetic components (n = 53, 63, 23, and 39, respectively). p values for statistical significance were calculated using Kruskal–Wallis test followed by a Dunn’s multiple comparisons test. ns, non-significant, p > 0.05; *, p < 0.05; **, p < 0.01; ***, p < 0.005; ****, p < 0.0001. (PDF) [file pgen.1011980.s006.pdf]

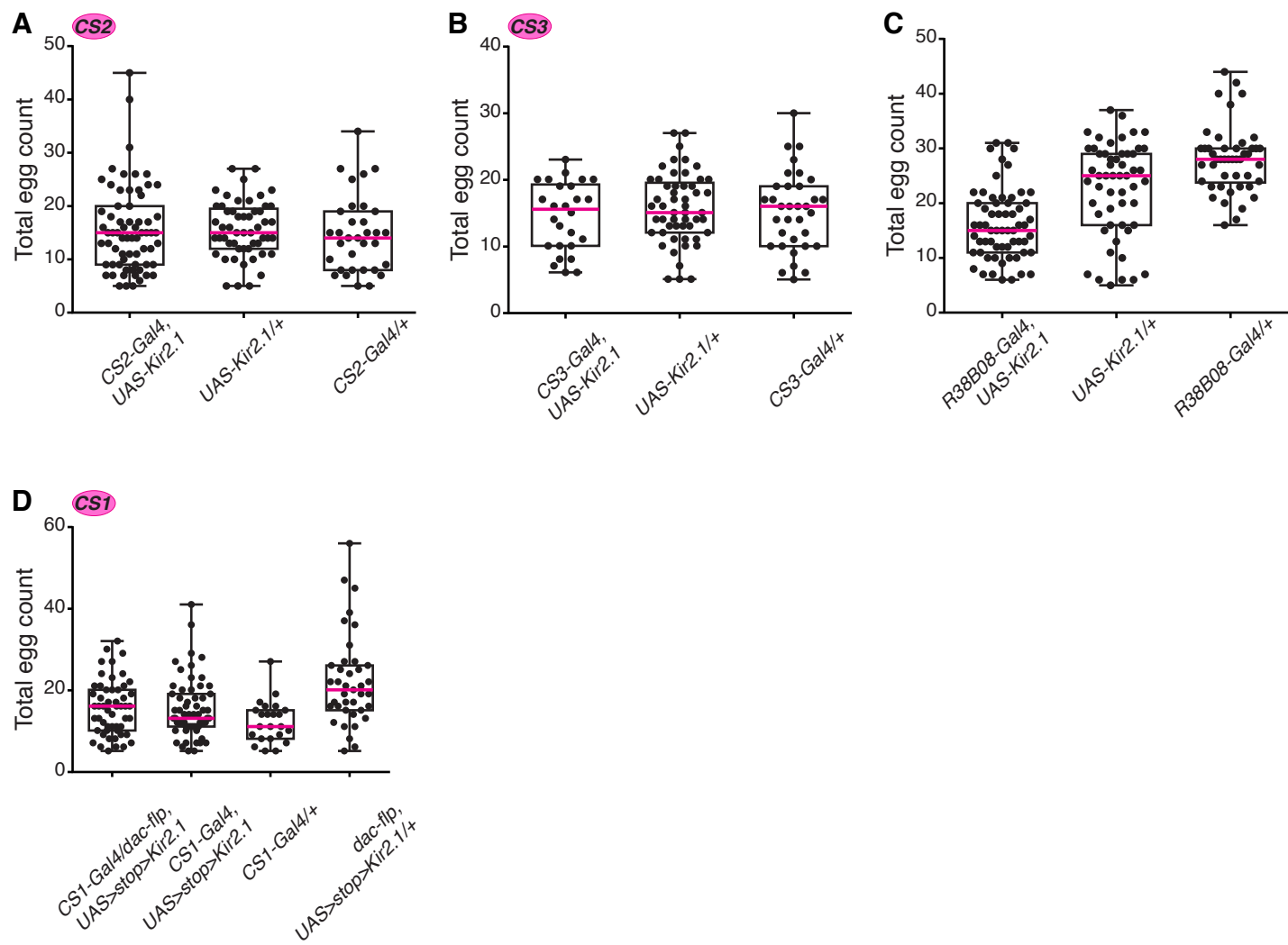

Supplement: S7 Fig — (A-C) Total number of eggs laid per mated female, involving Kir2.1-mediated silencing of neurons labelled by CS2-Gal4 (A), CS3-Gal4 (B) and R38B08-Gal4 (C), respectively. (D) Total number of eggs laid per mated female, involving conditional silencing of mechanosensory neurons innervating subsets of campaniform sensilla and mechanosensory bristles labelled by CS1-Gal4. (PDF) [file pgen.1011980.s007.pdf]

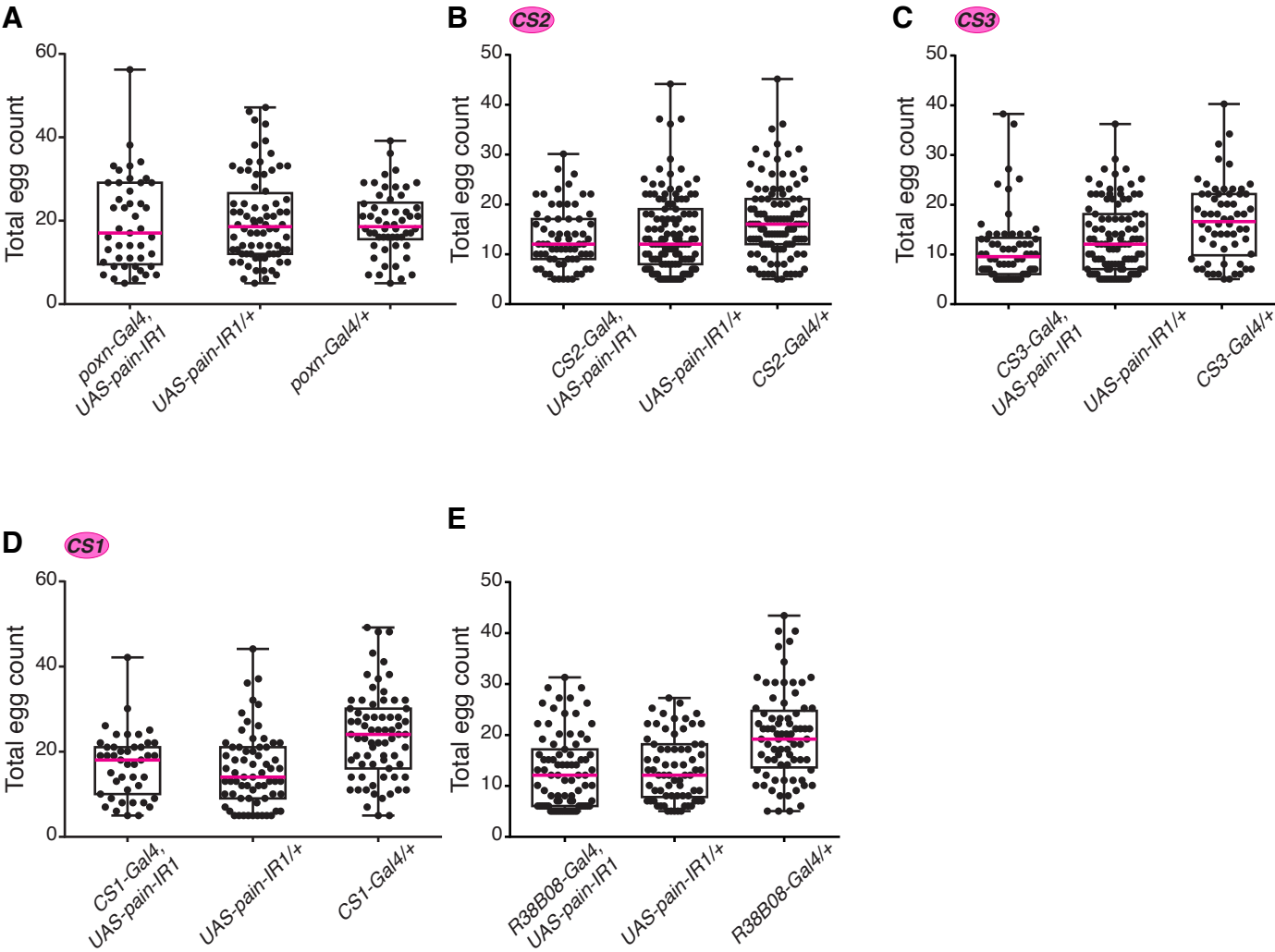

Supplement: S8 Fig — (A-E) Total number of eggs laid per single mated female upon knocking down pain expression (UAS-pain-IR1) in chemosensory neurons associated with chemosensory bristles labelled by poxn-Gal4 (A), mechanosensory neurons innervating subsets of campaniform sensilla labelled by CS2-Gal4 and CS3-Gal4 (B-D), both campaniform sensilla and mechanosensory bristles labelled by CS1-Gal4, and only mechanosensory bristles by R38B08-Gal4 (E), respectively. (PDF) [file pgen.1011980.s008.pdf]

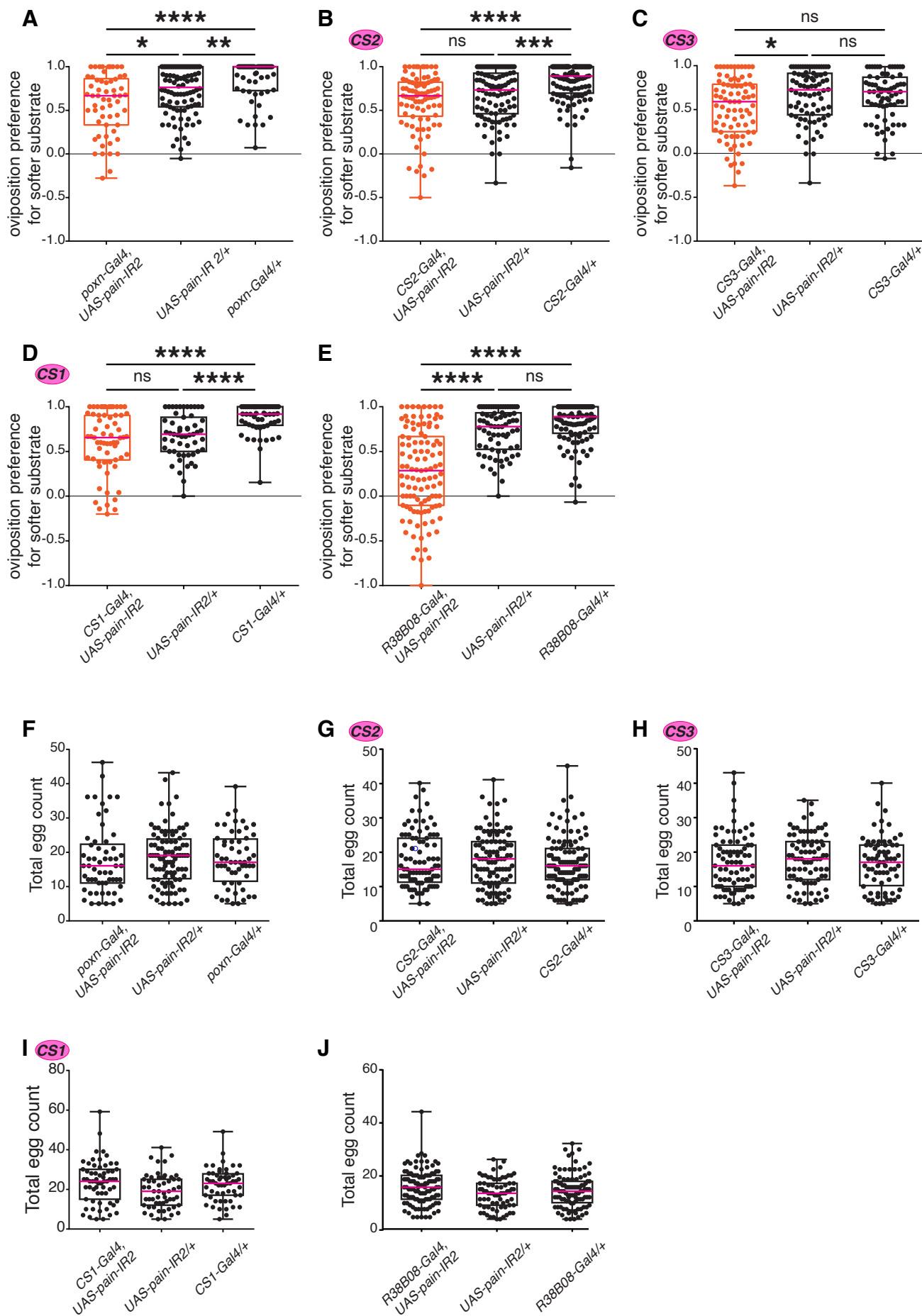

Supplement: S9 Fig — Single-fly two-choice stiffness assay (0.5% vs. 1.25% agarose) of females with RNAi-mediated down-regulation of pain expression in neurons innervating chemosensory bristles (A, F), campaniform sensilla (B-D, G-I), or mechanosensory bristles (E, J). (A) poxn-Gal4, UAS-pain-IR2, compared to UAS-alone or Gal4-alone controls (n = 58, 96, and 56, respectively). (B) CS2-Gal4, UAS-pain-IR2, compared to UAS-alone or Gal4-alone controls (n = 88, 99, and 112, respectively). (C) CS3-Gal4, UAS-pain-IR2, compared to UAS-alone or Gal4-alone controls (n = 80, 80, and 72, respectively). (D) CS1-Gal4, UAS-pain-IR2, compared to UAS-alone or Gal4-alone controls (n = 63, 55, and 56, respectively). (E) R38B08-Gal4, UAS-pain-IR2, compared to UAS-alone or Gal4-alone controls (n = 83, 70, and 77, respectively). (F-J) Total egg counts used to calculate preference indices in panels A-E. p values for statistical significance were calculated using Kruskal–Wallis test followed by a Dunn’s multiple comparisons test. ns, non-significant, p > 0.05; *, p < 0.05; **, p < 0.01; ***, p < 0.005; ****, p < 0.0001. (PDF) [file pgen.1011980.s009.pdf]

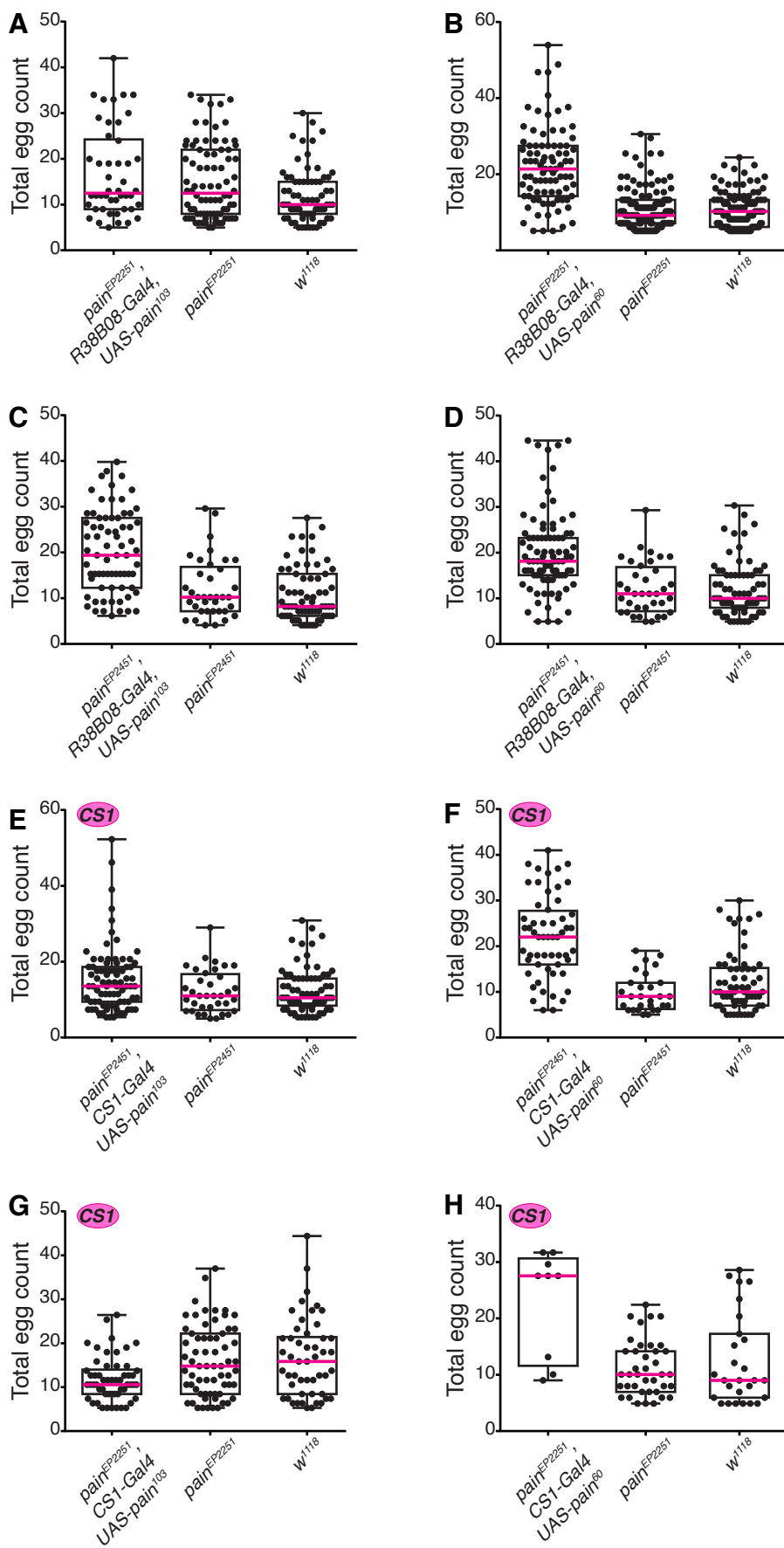

Supplement: S10 Fig — Total number of eggs laid upon overexpressing Painlessp103 (A, C, E, and G) and Painlessp60 (B, D, F, and H) isoforms using R38B08-Gal4 (A, B, C, and D), and CS1-Gal4 (E, F, G, and H) in pain [EP2251] (A, B, G, and H) and pain [EP4251] (C, D, E, and F) mutant backgrounds. (PDF) [file pgen.1011980.s010.pdf]
