# Supplementary material for: The TRP-channel painless mediates substrate stiffness sensing in the legs during Drosophila oviposition
Source: PLoS Genet. 2025 Dec 29;21(12):e1011980. doi: 10.1371/journal.pgen.1011980 (PMC12768414; doi:10.1371/journal.pgen.1011980)
Supplement: S11 Fig — Assembly of a single fly two-choice oviposition chamber, consisting of a lower and upper half. The divider (A) with walls of 0.5 mm is fitted on top of the base (B) containing 9 lanes into which alternatingly soft and hard substrates are poured, to create the lower half (C). This composite part provides 56 chambers, each containing patches of soft and hard substrates, separated by the divider. To construct the upper half, a spacer (E) is attached to the base of the loft (D), forming a composite unit (F). A porous lid cut out of 1.5 mm acrylic, with 2.8 mm diameter (circular loading ports) is then glued on top of it (G) to constitute the upper half of the behavioral apparatus (H). This upper half (H) is then placed on top of (C), and the entire apparatus is held in place with rubber bands. The final dimensions of an individual chamber is 8.4mm x 10mm (width (x) by length (y)) with a height of 8.4 mm (1 mm (base + divider) + 7.4 mm (spacer + loft). (PDF) [file pgen.1011980.s011.pdf]

**Ray, et al., Figure S11**

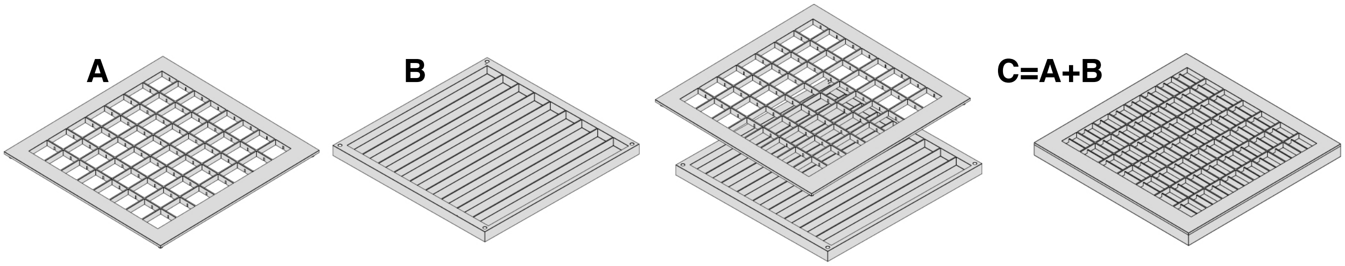


Making of lower apparatus

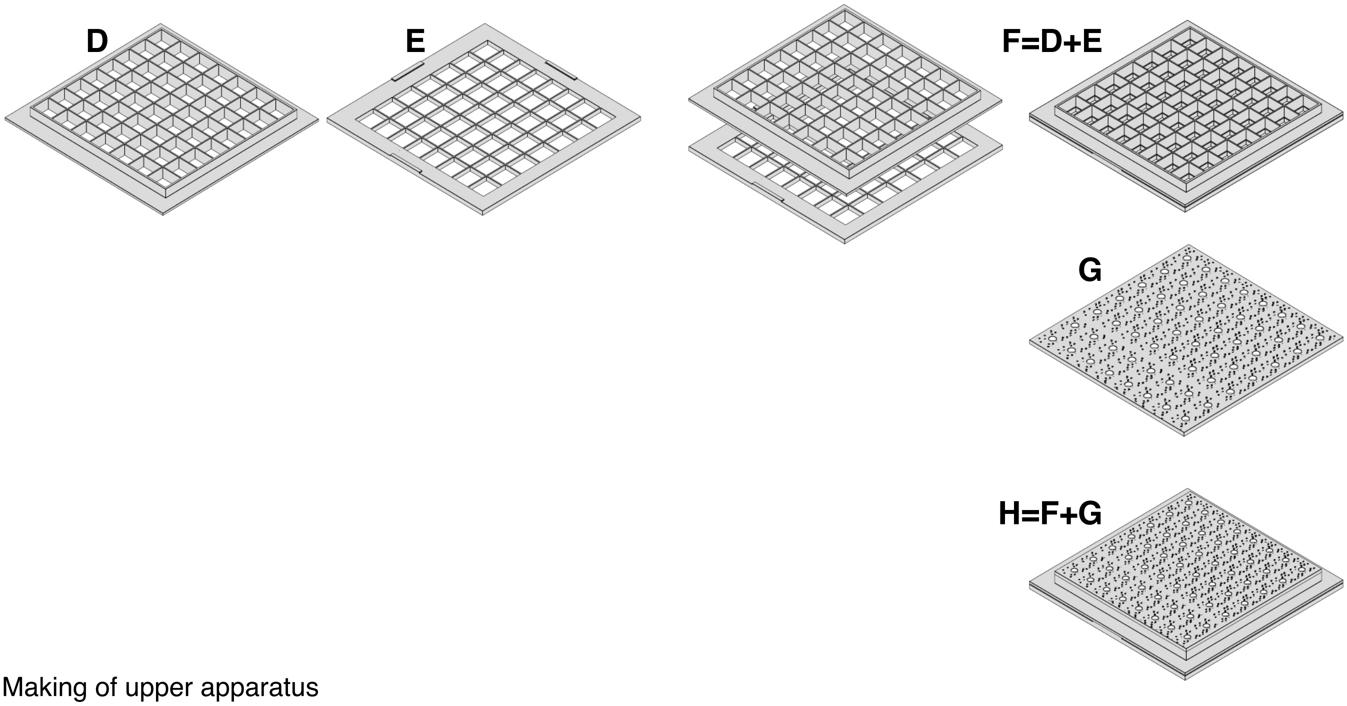


Making of upper apparatus

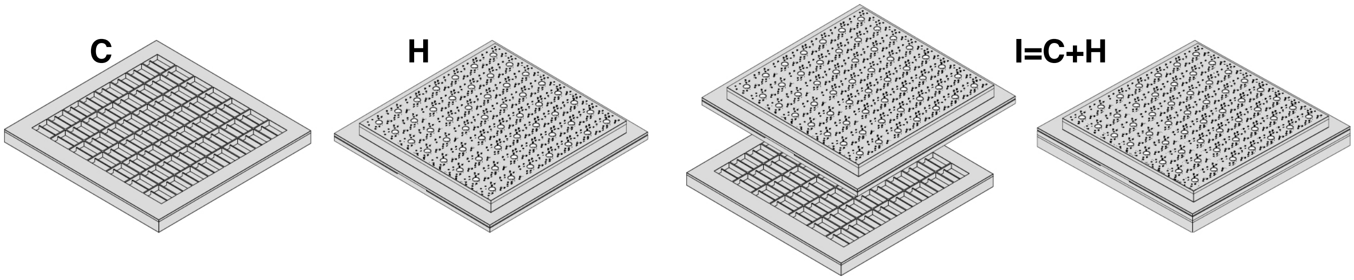


Assembly of lower and upper apparatus to construct the assay chamber
